# Supplementary material for: Psychometric properties of the Probability Bias Measure
Source: PLoS One. 2026 Apr 24;21(4):e0346803. doi: 10.1371/journal.pone.0346803 (PMC13108805; doi:10.1371/journal.pone.0346803)
Supplement: S1 File — (DOCX) [file pone.0346803.s001.docx]

**Psychometric properties of the Probability Bias Measure**

Robert W. Booth, Gubse N. Aydın, Beril Başara, and Ceren Yılmaz

# S-1

Here we present the Probability Bias Measure in both Turkish and English. For ease of comprehension, we present positive and negative events separately; note that events should be presented to participants one-at-a-time, in a random order. Note also that no numbers should be presented on the response scale; the responses can be coded with scores of 1 to 7 for analysis.

## Instructions

Bu ankette, size bazı durumlar tasvir edilecektir. Lütfen uygun cevabı seçerek her bir durumun sizin başınıza gelme olasılığını belirtmenizi rica ediyoruz. Doğru ya da yanlış cevap yoktur. Karşılaşacağınız tüm farklı olayların başınıza gelme olasılığını değerlendirirken dikkatinizi korumanız önemlidir. İlginiz için şimdiden teşekkür ederiz.

In this survey, we will describe a series of situations. Please indicate how likely each situation is to happen to you by choosing the appropriate response option. There are no right or wrong answers. Please keep focused while you are rating the likelihood of each event. Thank you for your attention.

## Response Scale

Bana asla olmaz

Muhtemelen bana olmaz

Bana olmayabilir

Bana olabilir ya da olmayabilir

Bana olabilir

Muhtemelen bana olur

Bana kesinlikle olur

Would never happen to me

Would probably not happen to me

Might not happen to me

Might happen, might not

Might happen to me

Would probably happen to me

Would definitely happen to me

## Positive Items

Olağanüstü bir başarı ile tanınacaksınız.

Alanınızda çok tanınan biri olacaksınız.

Hayatınızdan çok memnun olacaksınız.

Yarın sizin için harika bir gün olacak.

Yeni insanlarla tanışacak ve iyi bir izlenim bırakacaksınız.

Görünüşünüzle ilgili iltifat alacaksınız.

90. yaş gününüz harika olacak.

Çok enerjik ve coşkulu hissedeceksiniz.

Hedeflerinize ulaşacaksınız.

Çok zinde ve sağlıklı olacaksınız.

Stres ile kolayca başa çıkabileceksiniz.

Zihniniz çok dikkatli ve farkındalıklı olacak.

You will become well-known for an outstanding accomplishment.

You will be very well-known in your field.

You will be completely satisfied with your life.

Tomorrow will be a wonderful day for you.

You meet some new people, and make a good impression.

You receive compliments about your appearance.

You will have a wonderful 90th birthday.

You will have lots of energy and enthusiasm.

You will achieve the things you set out to do.

You will be very fit and healthy.

You will be able to cope easily with pressure.

Your mind will be very alert and 'on the ball'.

## Negative Items

Pişman olacağınız bir karar alacaksınız.

İnsanlar sizi sıkıcı bulacak.

İnsanlar sizi başarısız bulacak.

İşlerinizde çok geride kalacaksınız.

Kimse ile dertleşmeniz mümkün olmayacak.

Konuşurken aptal gibi hissedeceksiniz.

Zihniniz normal çalışmayı bırakacak.

Kötü performanstan dolayı eleştirileceksiniz.

İnsanların önünde telaşlı hissedeceksiniz.

Delireceksiniz.

Başkalarına kıyasla yetersiz hissedeceksiniz.

Sosyal ortamlarda kendinizi ifade edemeyeceksiniz.

You will make a decision which you later regret.

People will find you boring.

People will think you’re a failure.

You will fall badly behind in your work.

You will be unable to confide in anyone.

You will feel stupid while talking to others.

Your mind will stop functioning normally.

You will be criticised for poor performance.

You will feel flustered in front of others.

You will go crazy.

You will feel inferior to others.

You will be unable to express yourself in social situations.
