# Supplementary material for: Psychometric properties of the Probability Bias Measure
Source: PLoS One. 2026 Apr 24;21(4):e0346803. doi: 10.1371/journal.pone.0346803 (PMC13108805; doi:10.1371/journal.pone.0346803)
Supplement: S2 File — (DOCX) [file pone.0346803.s002.docx]

# Psychometric properties of the Probability Bias Measure

# Robert W. Booth, Gubse N. Aydın, Beril Başara, and Ceren Yılmaz

# Sabanci University, Istanbul, Turkey

# S2

Table S2

*Full list of Turkish events, as used in Study 1. Approximate English translations are provided.*

| **Turkish** | **English** |
| --- | --- |
| Önümüzdeki sene içinde cep telefonunuz kaybolacak veya ciddi şekilde hasar alacak | You will lose or seriously damage your mobile phone in the next year |
| Bir sonraki sosyal etkinliğinizde kendinizi utandıracaksınız | You'll embarrass yourself at your next social event |
| Önümüzdeki ay içinde aileniz ile ciddi bir tartışma yaşayacaksınız | You will have a serious argument with your family in the next month. |
| Önümüzdeki sene içinde sevdiğiniz birini kaybedeceksiniz | You will lose someone you love in the next year. |
| Şiddete maruz kalacaksınız | You will be subjected to violence |
| Hayatınızda, yalnız geçirdiğiniz uzun bir dönem olacak | You will spend a long period in your life alone. |
| Evsiz kalacaksınız | You will become homeless |
| Bir doğal afette ciddi şekilde yaralanacaksınız | You will be seriously injured in a natural disaster |
| Önümüzdeki 5 sene içerisinde ciddi bir araba kazası geçireceksiniz | You will have a serious car accident in the next 5 years |
| Bir arkadaşınızın bilgisayarını ödünç alsanız, yanlışlıkla ona zarar verirdiniz | If you borrowed a friend's computer, you would accidentally damage it |
| En yakın arkadaşınız sizi görmezden gelmeye başlıyor | Your best friend starts ignoring you |
| Küçük bir kazada yaralanıyorsunuz | You are injured in a minor accident |
| Sosyal medyada alay konusu oluyorsunuz | You are mocked on social media |
| Birkaç arkadaşınızın cumartesi günü buluşma planlayıp sizi davet etmediklerini öğreniyorsunuz | You find out that some of your friends are planning to meet up on Saturday but didn't invite you. |
| Kendinizi kırık bir camla kesiyorsunuz ve kötü bir enfeksiyon geçiriyorsunuz | You cut yourself on broken glass, and get a nasty infection |
| Bir gece evde yalnızken hırsızlar içeri giriyor | One night, when you are home alone, thieves will break in. |
| Bir otorite figürü tarafından haksızlığa uğruyorsunuz | You will be mistreated by someone in authority |
| Ciddi bir fiziksel hastalık teşhisi alacaksınız | You will be diagnosed with a serious physical illness |
| Bacaklarınızı kaybedeceksiniz ve tekerlekli sandalyeye mahkum kalacaksınız | You will lose your legs and be confined to a wheelchair. |
| İyi bir arkadaşınız ile ciddi bir anlaşmazlık yaşayacaksınız | You will have a serious disagreement with a good friend. |
| Pişman olacağınız bir karar alacaksınız | You will make a decision which you later regret |
| Anlaşılamadığınızı hissedeceksiniz | You will feel like you are not understood |
| Yanlış giden şeyler yüzünden suçlanacaksınız | You will be blamed for things that have gone wrong |
| Bir yakınınız tarafından reddedileceksiniz | You will be rejected by someone close to you |
| İşler umduğunuz gibi gitmeyecek | Things won't go as you hoped |
| İnsanlar sizden hoşlanmayacak | People won't like you |
| İnsanlar sizi sıkıcı bulacak | People will find you boring |
| İnsanlar sizi başarısız bulacak | People will think you’re a failure |
| Pek çok hata yapacaksınız | You will make many mistakes |
| İşlerinizde çok geride kalacaksınız | You will fall badly behind in your work |
| Kimse ile dertleşmeniz mümkün olmayacak | You will be unable to confide in anyone |
| Yorgun ve uyuşuk olacaksınız | You will be tired and lethargic |
| İnsanlar sizinle alay edecek | People will make fun of you |
| Bir yakınızı hayal kırıklığına uğratacaksınız | You will disappoint someone close to you |
| Sorumluluklarınızla baş edemeyeceksiniz | You will not be able to cope with your responsibilities |
| Konuşurken aptal gibi hissedeceksiniz | You will feel stupid while talking to others |
| Her şeyin ters gittiği bir gün geçireceksiniz | You will have a day where everything goes wrong |
| Tutuklanacaksınız | You will be arrested |
| Ciddi bir baş ağrınız olacak | You will have a severe headache |
| Bir pazarlamacı sizi arayacak | A telemarketer will call you |
| Tanımadığınız biri size bağıracak | You will be yelled at by a stranger |
| Bir randevu veya toplantıya 15 dakika veya daha fazla geç kalacaksınız veya tamamen kaçıracaksınız | You will be 15 or more minutes late for an appointment or meeting, or miss it entirely |
| Önemli bir son teslim tarihini unutacaksınız | You will forget an important deadline |
| Şahsen tanıdığınız birinin öldüğünü öğreneceksiniz | You will learn that someone you knew personally has died |
| Felç geçireceksiniz | You will have a stroke |
| Görme duyunuzu kaybedeceksiniz | You will lose your eyesight |
| Zihniniz normal çalışmayı bırakacak | Your mind will stop functioning normally |
| Hafızanızı kaybedeceksiniz | You will lose your memory |
| Zihinsel olarak engellenmiş hissedeceksiniz | You will feel mentally blocked |
| Evde sizi elektrik çarpacak | You will get an electric shock while at home |
| Kötü performanstan dolayı eleştirileceksiniz | You will be criticised for poor performance |
| Yatak odanızın yakınlarından gelen inşaat sesinden dolayı çok iyi uyuyamayacaksınız | You won't sleep very well due to construction noise coming from near your bedroom. |
| Yaşadığınız yer yarın çok bulutlu ve yağmurlu olacak | where you live, it will be very cloudy and rainy tomorrow |
| Önümüzdeki 10 yıl içerisinde öleceksiniz | You will die within the next 10 years |
| Bu kış grip olacaksınız | You will get the flu this winter |
| Kalp hastalığına yakalanacaksınız | You will get heart disease |
| Yaptığınız bir şeyden dolayı utanacaksınız | You will feel ashamed of something you did |
| İnsanların önünde telaşlı hissedeceksiniz | You will feel flustered in front of others |
| Bir partideyken insanlar endişeli olduğunuzu fark edecek | When you're at a party, people will notice that you're anxious |
| Bir iş görüşmesi veya değerlendirmesi sırasında donup kalacaksınız | You will freeze during a job interview or evaluation |
| Konuştuğunuz sırada bir kişi ortamdan ayrılacak | Someone will leave the room while you are speaking |
| Tanıdığınız bir kişi tarafından yok sayılacaksınız | You will be ignored by someone you know |
| Toplum içinde aptalca bir şey yapacaksınız | You will do something stupid in public |
| Önemli bir amacınıza ulaşamayacaksınız | You will not be able to achieve an important goal |
| Süpervizörünüz beklenmedik bir şekilde iş yerinde sizi görmek isteyecek | Your supervisor will unexpectedly ask to see you at work |
| Büyük bir cerrahi operasyon geçirmeniz gerekecek | You will need to undergo major surgery |
| Toplum içinde bayılacaksınız | You will faint in public |
| Bir yakınınıza ölümcül bir hastalık tanısı konulacak | A relative of yours will be diagnosed with a terminal disease |
| Cüzdanınızı kaybedeceksiniz | You will lose your wallet |
| Delireceksiniz | You will go crazy |
| Umumi tuvaletleri kullanmaktan dolayı hastalık kapacaksınız | You will get an infection from using a public toilet |
| Banka hesabınızın eksiye düştüğünü göreceksiniz | You will learn that your bank account is overdrawn |
| Evinizde yangın çıkacak | There will be a fire in your house |
| Bir konuşma esnasında ne söyleyeceğinizi bilemeyeceksiniz | You won't know what to say during a conversation. |
| İçeceğinizi devireceksiniz | You will spill your drink |
| Biri size karşı ukalalık yapacak | Someone will talk down to you |
| Küçük bir operasyon geçirmeniz gerekecek | You will need to undergo a minor operation |
| Göğsünüzde sebepsiz bir ağrı hissedeceksiniz | You will feel pain in your chest for no reason. |
| Ev anahtarlarınızı kaybedeceksiniz | You will lose your house keys |
| Başkalarına kıyasla yetersiz hissedeceksiniz | You will feel inferior to others |
| Sosyal ortamlarda kendinizi ifade edemeyeceksiniz | You will be unable to express yourself in social situations |
| Ön görülemeyen maddi sıkıntılar yaşayacaksınız | You will experience unforeseen financial difficulties. |
| Konuştuğunuz kişi sizi dinlerken esneyecek | The person you are talking to will yawn while listening to you |
| Bir arkadaşınız sizinle buluşma randevusunu iptal edecek | A friend will cancel an appointment to meet you. |
| Kazara bir şeyi kıracaksınız | You will accidentally break something |
| Hoşunuza gitmeyen insanlarla çalışmak zorunda kalacaksınız | You will have to work with people you don't like. |
| Olağanüstü bir başarı ile tanınacaksınız | You will become well-known for an outstanding accomplishment |
| Mutlu bir evliliğiniz olacak | You will have a happy marriage |
| Çok zengin olacaksınız | You will become very rich |
| Bir sonraki girişiminizde veya hedefinizde başarılı olabileceksiniz | You will be able to succeed in your next venture or goal. |
| Katılacağınız bir sonraki partide veya sosyal etkinlikte iyi karşılanacaksınız | You will be welcomed at the next party or social event you attend. |
| Düzenli olarak oynarsanız, piyangoyu kazanırsınız | You would win the lottery if you played regularly |
| Alanınızda çok tanınan biri olacaksınız | You will become very well-known in your field |
| Olağanüstü yetenekli bir çocuğunuz olacak | You will have an unusually gifted child |
| Yaşlılığınızda canlı ve sağlıklı olacaksınız | You will be alive and healthy well into old age |
| Hayatınızdan çok memnun olacaksınız | You will be completely satisfied with your life |
| Bir sonraki tatilinizden çok keyif alacaksınız | You will very much enjoy your next holiday |
| Bir kriz anında arkadaşlarınız sizi desteklemek için yanınızda olacak | Your friends will always be there for you in times of crisis |
| Aileniz her zaman sizinle gurur duyacak | Your family will always be proud of you |
| Yarın sizin için harika bir gün olacak | Tomorrow will be a wonderful day for you |
| Bir süredir hoşlandığınız birisi size kendisinin de sizden hoşlandığını söylüyor | Someone you've liked for a while tells you that they like you too |
| Arkadaşınız doğum gününüz için bir sürpriz planlıyor | Your friend is planning a surprise for your birthday |
| Yeni insanlarla tanışıyorsunuz ve iyi bir izlenim bırakıyorsunuz | You meet some new people, and make a good impression |
| Bir rekabeti veya yarışmayı kazanıyorsunuz | You win a competition or contest |
| Görünüşünüzle ilgili iltifat alıyorsunuz | You receive compliments about your appearance |
| Bir arkadaşınız sizi kendini savunma dersine götürüyor ve bunda oldukça iyi olduğunuzu keşfediyorsunuz | A friend takes you to a self-defence class, and you discover that you're rather good at it |
| Yeni istatistikler yaşadığınız bölgede suç oranının düşmekte olduğunu gösteriyor | New statistics show crime rates are falling in your area |
| 6 ay boyunca soğuk algınlığı geçirmiyorsunuz | You go six months without catching a cold |
| 90 yaş gününüz harika olacak | You will have a wonderful 90th birthday |
| İnsanlar size hayranlık duyacak | People will admire you |
| Çok enerjik ve coşkulu hissedeceksiniz | You will have lots of energy and enthusiasm |
| Hedeflerinize ulaşacaksınız | You will achieve the things you set out to do |
| Çok zinde ve sağlıklı olacaksınız | You will be very fit and healthy |
| Arkadaşlarınızla çok güzel zamanlarınız olacak | You will have a great time with your friends. |
| Stres ile kolayca başa çıkabileceksiniz | You will be able to cope easily with pressure |
| Zihniniz çok dikkatli ve farkındalıklı olacak | Your mind will be very alert and 'on the ball' |
| İyi ve uzun süreli arkadaşlıklar kuracaksınız | You will make good and long-lasting friendships. |
| Kaybettiğiniz bir eşyayı bulacaksınız | You will find an item you lost |
| Yarışmaya dayalı bir sporu veya oyunu kazanacaksınız | You will win a competitive sport or game |
| Şehir dışında yaşayan bir arkadaşınız sizi ziyaret edecek | You will be visited by a friend from out of town |
| Bir süpervizör veya öğretmen yaptığınız işi övecek | A supervisor or instructor will praise your work |
| Bir partiye davet edileceksiniz | You will be invited to a party |
| Uzun zamandır görmediğiniz bir arkadaşınızla karşılaşacaksınız | You will meet a friend you haven't seen for a long time. |
| Bir arkadaşınız sizi bir aktiviteye davet edecek | A friend invites you to an event |
| Bir akrabanız bir yarışma veya ödül kazanacak | A relative of yours will win a contest or prize |
| Birisi size bir şov veya etkinlik için fazladan bir bilet verecek | Someone will give you an extra ticket to a show or event |
| Uzun süredir aradığınız bir eşyayı sonunda yeni bir dükkanda bulacaksınız | In a new shop, you will finally find an item you have been looking for |
